# Bariatric Artery Embolization for Obese Patients. An Up-to-Date Review

**DOI:** 10.5334/jbsr.3170

**Published:** 2023-09-26

**Authors:** Paolo Ravetta, Touda Kebbou, Mathilde Poras

**Affiliations:** 1 Department of Radiology, University Hospital Brugmann, Brussels, BE; 2 ULB, BE; 3 Department of Radiology, University Hospital Ibn Rochd, Casablanca, MA; 4 Department of Digestive Surgery, Saint-Pierre University Hospital, Brussels, BE

**Keywords:** bariatric artery embolization (BAE), left gastric artery embolization (LGAE), bariatric embolization, obesity, bariatric

## Abstract

Overweight and obesity are one of public health’s major problems in the world. Conservative treatment with exercise, diet and pharmacotherapy is often ineffective, especially in the long term. Bariatric surgery is the gold standard method for a sustained long-term weight loss. Recently the endovascular technique of bariatric artery embolization (BAE) has been studied as an obesity and overweight treatment, with promising results.

The goal of this article is to analyze the rationale behind BAE and to provide an up-to-date analysis of its strengths and limitation in comparison with bariatric surgery as a treatment for obesity.

## Introduction

During the last decades, overweight and obesity have been one of public health’s major problems in the world, affecting adults and children alike. Defined as having a body mass index (BMI) > 30 kg/m^2^, obesity is a major cause of morbidity and mortality, and it is a known risk factor for multiple diseases, including type 2 diabetes, cardiovascular diseases and cancer. In Belgium, referring to the 2018 Sciensano health report, 49.3% of the Belgian adult population is overweight (BMI ≥ 25) and 15.9% is obese; 19.0% of Belgian children (2–17 years) are overweight and 5.8% are obese [1].

Conservative and first-line treatments for overweight and obesity, including life nutritional rules, exercise and pharmacotherapy, can lead to a caloric deficit and a significative weight loss, but unfortunately those benefits are often temporary and not sustained long term [2,3].

Bariatric surgery is recognized by National Institute of Health and supported by quality evidence-based literature as the most effective method of long-term weight loss. In general, surgery, invasive in nature, is correlated with a high price and limited by a significant rate of morbidity and mortality [3,4]. Consequently, less invasive treatments that could also target the early stages of the disease are warranted.

Recently, the endovascular technique of bariatric artery embolization (BAE) has been studied as an obesity and overweight treatment with promising results [4]. Previously lacking quality articles, the literature has recently been completed by studies reviving the debate of its role in obesity management.

The goal of this article is to analyze the rationale behind BAE and to give an up-to-date analysis of its strengths and limitation in comparison with bariatric surgery as a treatment for obesity.

## Materials and Methods

A literature search was performed using PubMed, Scopus, Science Direct and Cochrane databases for studies which matched the eligibility criteria using the keywords ‘bariatric’, ‘embolization’, ‘BAE’ and ‘LGAE’. An additional manual search of bibliographies of each included study was done to identify studies not yet covered. Literature was consulted between 10 January 2021 and 3 January 2023.

## Results

### 1. Physiology, anatomy and rationale

The stomach, in addition to being an organ that serves to store, mix and digest food, can also be considered an organ that produces hormones, endocrine, paracrine and neurocrine. It is the primary site of production of gastrin via G cells and somatostatin via D cells in the antrum, histamine via enterochromaffin-like cells [5,6].

Ghrelin, a 28-amino acid peptide, plays a fundamental role in stimulating appetite and promoting positive energy balance to gain weight via activation of hypothalamic neurocircuits [3,7,8]. To date, ghrelin is the only orexigenic hormone identified, and 90% of the ghrelin-producing cells of the body are the X/A endocrine cells of the stomach fundus [9,10,11,12]. Mainly produced when the stomach is empty, which triggers feelings of hunger, ghrelin plasma levels in obese patients fail to downregulate after eating and have been found in high concentrations [6,13].

At present, all types of bariatric surgeries (including the most common ‘gastric bypass’ and ‘sleeve gastrectomy’) affect the antrum of the stomach, primarily by reducing its size but also by acting on the metabolism of hunger and satiety by decreasing the production of hormones acting on it, in particular ghrelin, PYY and GLP-1. This synergic action is the key behind patients’ weight loss, hence the name of ‘metabolic surgery’ [10,14].

In typical anatomy, the stomach gets its vascular supply from branches of the coeliac trunk. The lesser curvature, fundus and part of the anterior and posterior surfaces of the stomach are supplied by the left gastric artery, which is the smallest branch of the coeliac trunk. In a circle of anastomosis, the inferior part of the lesser curvature and the antrum are supplied by the right gastric artery, which usually originates from the proper hepatic artery. The greater curvature is supplied by the right and left gastroepiploic arteries, originating from the gastroduodenal artery and splenic artery, respectively. This anatomical division is the most common, though many variations may be possible. All these arteries make along their course many anastomoses to form a diffuse network to ensure an optimal supply to the stomach [10,15].

**Figure 1:**
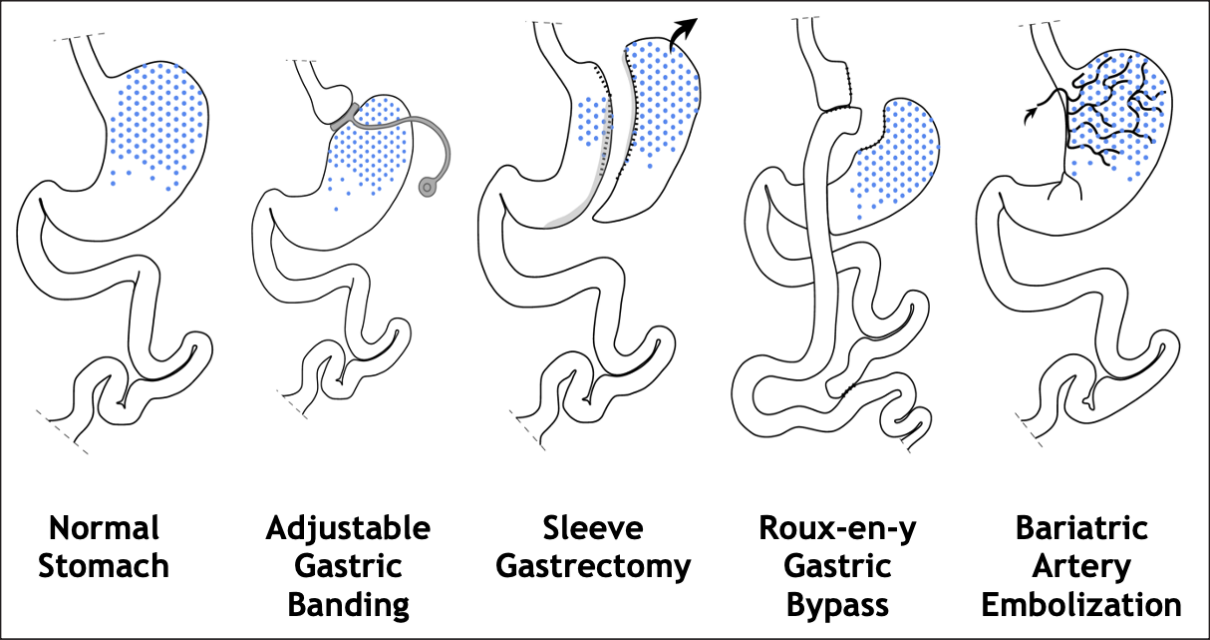
Anatomy and impact of surgery and bariatric embolization of the gastric fundus.

**Figure 2:**
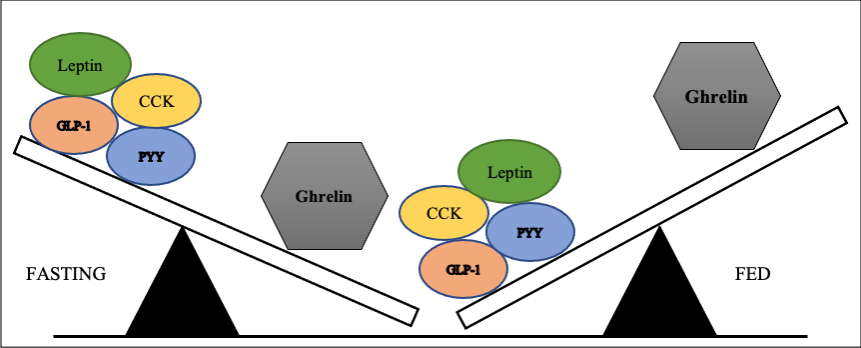
Hormonal balance theory during fasting and fed state.

BAE is a relatively recent procedure that has been developed. It is an alternative to the more invasive bariatric surgeries in order to minimize morbidity and hospital stay [16,17]. As ghrelin was described as an orexigenic hormone produced in the fundus, BAE was originally proposed as a minimally invasive, image-guided and percutaneous technique performed by interventional radiologists to modulate ghrelin production in the gastric fundus [3]. By embolizing the arteries supplying the gastric fundus, BAE aims to cause controlled, mild ischemic damage on the rapid turnover mucosa, thereby leading to reduced ghrelin production and promoting weight loss [2,3,17,18]. For hormonal balance and visual impact on surgery and BAE on gastric fundus, see Figures 1 and 2.

### 2. Procedure

Different techniques have been described for BAE, including access, microcatheters and embolic agents [19]. Most trials used a femoral approach [2,14,18,20,21]; only one, Pirlet et al. [22], used a radial approach. Left gastric artery is the most frequently embolized artery, with fewer studies selecting the gastroepiploic artery [18]. The most frequently used microcatheters varied between 2.7 F and 2.9 F.

Embolic agents used did not vary much. For example, Kipshidze et al. and Syed et al. used 300–500 µm microspheres [19,21]. Bai et al. used 500–710 µm polyvinyl alcohol particles [23]. Elens et al. and Weiss et al. used 500–700 µm embospheres [2,18].

Various studies reported data concerning radiation exposure and procedural time [16]. The average radiation dose reported by Syed et al., Weiss et al. and Pirlet et al. were 1369 mGy (ranging 128–2690), 5255 +/– 1662 mGy, and 187 +/– 113 mGy, respectively [15,21,22]. The total procedure time was only reported by Pirlet et al. and averaged 24 +/– 13 minutes [22]. Pirlet et al. analyzed the time and radiation needed to catheterize the LGA after performing coronarography, using a radial access, and reported a mean time of 7 (5–10) minutes, fluoroscopy time 160 (103–248) seconds, contrast volume 41 mL (33–63 mL) and a mean radiation exposure of 3125 (1906–4735) Gy.cm2 [22].

### 3. Results on weight loss

Weight loss has been the main focus of studies aiming to prove the safety and efficacy of BAE for humans. Since the first prospective human study of BAE performed specifically for weight loss was conducted by Kipshidze et al., numerous studies have proven the effectiveness of this procedure. A recent meta-analysis conducted by Hafezi et al. on 47 patients over six prospective clinical trials showed a mean weight loss of 8.68 kg +/– 1.24 kg (*p* < 0.001) (approximately 8% of baseline total body weight), after an average follow-up time period of twelve months after BAE [16,19]. The longest follow-up at two years after BAE was reported in the recent study of Pirlet et al. and in the randomized controlled trial of Reddy et al. The first study showed a mean weight loss of 7.7% (IQR, 3.2%–14,1%) and an absolute weight loss of 11 kg (IQR 4.25–21.5 kg). Reddy et al. showed a mean weight loss of 9.0% [24,25]. In the prospective study of Levigard et al., ten patients lost 6.8% (6.22 kg ± 3.6; *p* = 0.01) over a period of six months [6]. In Zaitoun et al., all patients decreased their body weight with a mean loss of 8.9% at six months [26]. Principals’ studies and outcomes are listed in Table 1.

**Table 1:** Weight loss after bariatric artery embolization in prospective or RC trials.


FIRST AUTHOR[REFERENCE]	YEAR	DESIGN	BASELINE BMI	AGE, Y	N° PATIENTS	FOLLOW-UP, MONTH	ABSOLUTE WEIGHT LOSS, KG	MEAN WEIGHT LOSS, %

Kipshidze [20]	2015	prospective	42.2 ± 6.8	44.7 ± 7.4	5	20–24	22.4 ± 4,2	17.19

Syed [21]	2016	prospective	42.4 ± 2.5	41.0 ± 10,7	4	6	9.19 ± 6.0	8.52

Bai [23]	2017	prospective	38.1 ± 3.8	42.8 ± 13.9	5	9	12.90 ± 14.7	12.64

Weiss [18]	2019	prospective	45 ± 4.1	44 ± 11	20	12	7.62	11.5

Elens [2]	2019	prospective	28.9 ± 2.5	38 ± 10	11	6	8.00 ± 5.1	10.00

Zaitoun [26]	2019	prospective	37.4 ± 3.3	37.5 ± 8.8	10	6	8.24 ± 3.1	8.90

Reddy [25]	2020	RCT	39.6 ± 3.8	45.5 ± 9.8	20	24	NA	9.00

Pirlet [24]	2020	prospective	49.4 (IQR 43.2–61.7)	NA	7	24	11 (IQR 4.25–21.5)	7.7 (IQR 3.2–14.1)

Levigard [27]	2021	prospective	36.4 ± 2.6	37.5 ± 7.3	10	6	6.22 ± 3.6	6.8


Age, baseline BMI are reported as mean ± SD (standard deviation) or mean (IQR (interquartile ranges)). BMI: body mass index; NA: not available; RCT: randomized controlled trial.

### 4. Results on lowering ghrelin levels

Concerning the effectiveness in lowering the blood concentration of ghrelin, the ghrelin reduction percentage was lowered by 41% at three months; 24.8% at nine months in Bai et al.; 36% at three months; and still 21% below baseline at twelve months in Kipshidze et al. [20,23]. In Levigrad et al., there was a sustained and continuous reduction of ghrelin levels reaching 32.7% at six months post-procedure [27]. In the BEAT Obesity trial, BAE was associated with ghrelin reduction. Persistent low ghrelin values up to twelve months post BAE were associated with significantly increased weight loss [28].

In some studies, serum leptin levels were also evaluated. Syed et al. reported a slight decrease of 24.1% (26 ng/mL vs 19.6 ng/mL) at six months. In Bai et al., a continuous decrease was observed, with a loss of 11.22% at nine months. In both studies, however, no statistical inference could be drawn due to their limited sample size.

GLP-1 hormone is also responsible in the regulation of food intake and energy expenditure; it has been shown to increase in the post–bariatric surgery hormonal changes [29]. In animal studies, GLP-1 has also been shown to increase, but no present human studies analyzing this aspect have found a statistical difference [28,30].

### 5. Results on glycemic control and lipidic profile

The effect of BAE on lowering lipid profile, HgA1C and metabolic syndrome has been investigated in some studies with promising results [16,19]. Zaitoun et al. showed that all patients decreased their HbA1c levels, with a mean loss of 21.4% at six months [26]. Levigrad et al. studied HbA1c, triglycerides, low-density lipoprotein (LDL) and high-density lipoprotein (HDL) levels. HbA1c decreased by 27,9% (from 6.58% ± 1.72 to 4.74% ± 2.58 (*p* = 0.06)), improving in patients with T2D at six months. Triglycerides, LDL and HDL showed no statistically significant differences [27]. Sayed et al. showed that HbA1c decreased from 7.4% to 6.3% (reduction of 14.9%) at three months, remaining there at six months [21]. In the BEAT Obesity trial [18], total cholesterol and LDL levels were lower at twelve months than their respective means at baseline (*p* = 0.08). Mean triglycerides and HDL decreased but then increased back. Hemoglobin A1c at twelve months was lower than at baseline (*p* = 0.047).

### 6. Complications

After reviewing human studies of BAE, a few minor adverse effects have been noted. The most frequent complications were not severe, such as mild nausea, transient vomiting and abdominal discomfort [2,18,19,22,23,24,25]. Fewer cases of superficial ulcers that were treated with proton pump inhibitors were observed [2,9,31]. Four cases of severe complications were reported. In the Elens et al. study, one patient developed pancreatitis associated with splenic infarction and gastric perforation [2]. Kim et al. noted gastric ulcer in two patients and jejunal ischemia in one patient. One fatal outcome was reported but was related to the medical history of the patient [31]. Puncture-related complications, like left distal radial artery thrombosis and hematoma of the puncture site, have been mentioned [23,27].

### 7. Bariatric surgery

Bariatric surgery is recognized by National Institute of Health and supported by quality evidence-based literature as the most effective method of long-term weight loss. The undisputed indications to benefit from surgery are a BMI > 40 kg/m^2^ or > 35 kg/m^2^ associated with diabetes, hypertension or sleep apnea [32].

The most frequently used interventions are sleeve gastrectomy (SG) and Roux-en-Y gastric bypass (RYGB). Other surgeries are available like adjustable gastric band (AGB), one-anastomosis gastric bypass (OAGB), biliopancreatic diversion or bipartition procedure, which is a new type of technique [10,33]. Almost all surgeries are done laparoscopically (up to 99.3% in 2018), and patients stayed less than three days at the hospital [33].

Although there is heterogeneity among studies, consistent studies reported an average percentage weight loss for all types of surgery at 28.9%. The first three years after RYGB is up to 30% to 35% of total body weight, 29% after SG and 16% of total body weight after AGB [17,33,34,35].

Surgical procedures were previously considered to function only as: restrictive because the size of the gastric pouch was greatly reduced; malabsorptive in which malabsorption of nutrients contributes to weight loss, and a combination of restrictive and malabsorptive components. There is now growing evidence suggesting that the gut hormone levels after bariatric surgery are, in part, responsible for the postprocedural weight loss [10,34]. This is why bipartition procedures have recently appeared: part of the food passes rapidly from the stomach or the duodenum into the terminal ileum, which immediately secretes satiety hormones (GLP-1, PYY-36) and leads to malabsorption.

Bariatric surgery contributes to improvement in metabolic comorbidities, especially RYGB. For example, following their weight loss, most patients will be able to reduce or stop their hypertension treatment. There is also better control of the lipid profile and a decrease in sleep apnea. Concerning type 2 diabetes, more than half of the patients will maintain a remission of diabetes after surgery [33,35].

Bariatric surgery, even when less invasively performed laparoscopically, remains a surgical intervention with a risk of early and late morbidity. Perioperative and postoperative mortality rates are generally low: 0.07% and 0.21% for laparoscopic adjustable gastric banding; 0.29% and 0.34% vertical SG; 0.38% and 0.72% for RYGB; 0.76% for open procedures; and 1.11% for laparoscopic procedures for biliopancreatic diversion with duodenal switch [36].

The nature and prevalence of complications depends on the surgery technique. Although known and manageable, they sometimes lead to surgical reintervention. With RYGB, serious complications occur in 0–37% of all patients, with around a 2%–13% rate of reoperations. Bariatric surgery is therefore effective on weight loss and offers patients better control of their metabolic comorbidities, but at the cost of surgical complications.

## Discussion

Overweight and obesity is a global health issue that affects adults and children and has many morbidities [37]. Throughout the years, various treatments have been used to deal with this issue. Conservative and first-line treatment for obesity, including life nutritional rules, exercise and pharmacotherapy, often lead to unsustainable weight loss [2,3].

Bariatric surgery has been the gold standard in treating obese patients to decrease their weight [38]. The pathophysiology of weight loss has been explored for those patients, as well as the many factors that contribute to it: a combination of malabsorption and, more importantly, hormonal changes that occur even when using different techniques, like RYGB or SG [39]. An increase in incretin (GLP-1 and PYY) levels was detected, while a decrease in ghrelin levels was seen. Ghrelin, 90% produced in the fundus, is an orexigenic hormone; therefore, its decrease leads to a lower appetite and a faster satiety. Incretins like GLP-1 and PYY are produced in the presence of nutrients in the intestinal lumen. Since nutrients arrive faster to the intestine after surgery, their production is more stimulated. They act on gastric emptying by slowing it, which favors satiety and improves glycemia. These hormonal changes are observed only a few days after the surgery, long before weight loss. However, when compared to hormonal levels during a diet-induced weight loss, the opposite was noted. Ghrelin levels were higher, data about GLP-1 levels was controversial and there was a decrease in PYY levels. This could explain the lower efficiency of diets in comparison to bariatric surgery [40].

Bariatric surgery is invasive, has a high cost and is prone to a significant rate of morbidity and mortality [3,4]. Consequently, less invasive treatments that could also target the early stages of the diseases are warranted. BAE aims to achieve results that are as efficient as surgery but with the least possible complications because it is minimally invasive and requires a shorter hospital stay.

### 1. BAE’s contribution to the management of obesity

It is important to restate that BAE is a procedure not intended to replace bariatric surgery, but as a complementary method to facilitate weight loss with lifestyle modification. To fill the existing gap for patients whose lifestyle modifications alone have not been successful, but for whom surgery is not a suitable option, embolization could serve as a bridge to bariatric surgery or as complementary option to bariatric surgery [13,16].

Some authors have expressed concerns about the safety of surgery following BAE, referring to this procedure as a ‘dead-end procedure’ because embolizing the gastric arteries could leave no room for the most effective bariatric surgeries, like RYGB and SG [41]. For example, in SG, where division of the short gastric arteries is routinely performed, the LGA is left as the primary vessel of the esophageal junction. Further studies need to clarify this aspect, but recent studies show promising results in this regard. One study reported an uneventful SG surgery three years post BAE [42]. In Reddy et al., two patients underwent a bariatric surgical intervention (SG and laparoscopic gastric plication) post BAE to further leverage weight loss and experienced no perioperative or postoperative complications [25].

Authors reported no signs of adhesion or any alteration of the serosal layer; only a more pronounced serpentine appearance of the small arteries in the serosal layer regarding the lesser curvature was observed.

Interestingly, there is a case reported of double BAE in an 18-month span [24]. During the second embolization, which was completed successfully without major complications, a re-vascularization of the LGA was noted.

Finally, as already stated in the literature, it is important to note that BAE does not imply irreversible occlusion and should allow for secondary embolization and surgical interventions [24]. This observation also paves the road for possible repeated embolization procedures with the goal of perpetuating or increasing weight loss.

### 2. Weight loss and hormonal changes

After bariatric surgery, consistent studies reported an average percentage weight loss for all types of surgery at 28.9%. On the contrary, since there have not yet been studies that analyze the long-term effects of weight loss after BAE, longtime follow-up data is warranted. However, recent reports have shown a consistent loss of an average weight of about 8%–9% (ranging 4.8%–17.2%) at one year [16]. The only RCT from Reddy et al. showed a mean loss of 9% at two years post-embolization [25].

Not uncommonly in bariatric procedures after a rapid weight loss, patients have shown a tendency to regain weight. This is probably the reflection of the hormonal changes. Although postoperative ghrelin has been reported to statistically drop by as much as 41%, it has been shown to further trend toward its original values [16]. Studies with bigger cohorts are needed to further evaluate not only the effectiveness of BAE in lowering the blood concentration of ghrelin in the long term but also the impact of the ghrelin reduction in weight loss. Changes in other hormones like GLP-1, proven to promote weight loss and implied in post–bariatric surgery’s hormonal changes, could also support the endocrinological rationale of BAE. Although a few studies analyzed GLP-1 changes post BAE, no statistical difference has been reported yet.

### 3. Selection of BAE candidates

Most studies are relatively recent and not standardized. Many hypotheses are still to be explored regarding the pathophysiology of obesity itself to properly determine the exact effect of embolization on weight loss [43]. Further studies need to be conducted to provide insights on the exact effect of BAE on lipidic profile, diabetes and metabolic syndrome. Increasing knowledge could be beneficial to develop more precise techniques and guidelines and to ultimately obviate whether BAE should be combined with other weight loss therapies [19,43].

Finding the ideal candidate for BAE is challenging. The main trials and prospective studies had substantial differences in patient selection. For example, the patients’ BMI in the GET LEAN and BEAT Obesity trials was no less than 40 kg/m^2^. It was no less than 30 kg/m^2^ for patients included in the Bai et al. trial. In Reddy et al., the mean BMI was 39.6 kg/m^2^. Interestingly, in the Elens et al. study, overweight (but not obese) patients also showed a marked effect of BAE. Still, as the results were similar and in limited cohorts, it is difficult to select the ideal patient’s BMI.

Some guidelines for patient selection from various radiological societies have recently been published [44,45]. Those guidelines agree on multidisciplinary care and to mainly advise BAE for patients with class II or III obesity who are ineligible for or (by personal preference) who choose not to have bariatric surgery. Recurrence among patients after bariatric surgery, and patients in end-stage liver disease who are not suitable candidates for transplant surgery due to class III obesity, have also been considered.

BAE has shown some significant effect on lowering HbA1c and the lipidic and glycemic profile [27], and some studies prefer this as the assessment of the primary outcome [27]. However, further studies are required to carefully evaluate these changes.

## Conclusion

To conclude, bariatric artery embolization (BAE) is still a relatively new but promising technique. Although the results in weight loss are less important than after bariatric surgery, and there are no studies analyzing the long-term effects of weight loss after BAE, it has been shown to be a safe and effective procedure.

Many advantages have been equally demonstrated in terms of decrease of obesity comorbidities, such as diabetes and lipidic profiles. This paves the road for further developments in this technique and its potential application.

However, there are still numerous questions that need to be explored concerning its indications, the appropriate material, approaches to use and parameters for objective results in the long term. Randomized controlled trials using stratified patient sampling could also determine its effectiveness and compare it to the actual gold standards used for weight loss, like RYGB and SG.
